# Supplementary material for: Prevalence and mechanisms of evolutionary contingency in human influenza H3N2 neuraminidase
Source: Nat Commun. 2022 Oct 28;13:6443. doi: 10.1038/s41467-022-34060-8 (PMC9616408; doi:10.1038/s41467-022-34060-8)
Supplement: Supplementary file 1 — Supplementary Information [file 41467_2022_34060_MOESM1_ESM.pdf]

Supplementary Table 1. X-ray data collection and refinement statistics.

	Mos99 NA	Bil69 NA	SD93 NA + Zanamivir
**Data collection**			
Wavelength (Å)	0.97856	0.97872	1.12723
Resolution (Å)	1.397	1.537	1.645
Resolution Range [a]	43.458-1.397 (1.401-1.397)	46.049-1.537 (1.563-1.540)	76.340-1.700 (1.650-1.645)
Space group	I 4 2 2	C 2	P 4 2_1 2
Cell dimensions			
a, *b*, *c* (Å)	136.15, 136.15, 150.77	116.34, 137.94, 138.25	107.95, 107.95, 78.62
α, β, γ (°)	90.00, 90.00, 90.00	90.00, 92.16, 90.00	90.00, 90.00, 90.00
Total reflections	3,233,337	2,400,688	520,061
Unique reflections	138,645	322,494	54,981
Multiplicity [a]	23.0 (14.7)	7.5 (7.5)	9.5 (6.6)
Completeness (%) [a]	99.9 (99.2)	100.0 (100.0)	96.8 (97.3)
$<I/\sigma_I>$ [a]	20.2 (2.2)	12.1 (2.1)	11.5 (2.1)
R_{merge} (%) [a]	10.8 (126.0)	10.4 (98.9)	12.7 (77.4)
R_{meas} (%) [a]	11.3 (130.7)	11.1 (106.4)	13.8 (83.4)
$CC_{1/2}$ [a]	0.999 (0.674)	0.998 (0.778)	0.998 (0.895)
**Refinement**			
Resolution (Å)	43.46-1.40	25.00-1.54	25.00-1.65
No. reflections	138,634	304,302	51,828
R_{work} [c] / R_{free} [d]	0.151/0.164	0.166/0.180	0.188/0.215
No. atoms			
Protein	3,055	12,156	3,016
Water	583	2,004	317
Sugars/Inhibitor	124	408	149
B-factors			
Protein	14.2	14.9	18.0
Sugars/Inhibitor	25.1	25.5	33.1
Water	30.8	29.8	26.2
**RMSD from ideal geometry**			
Bond lengths (Å)	0.007	0.003	0.005
Bond angles (°)	1.12	0.995	1.210
**Ramachandran statistics (%)**			
Favored	99.7	95.7	96.1
Outliers	0.00	0.00	0.00
**PDB code**	7U4F	7U4E	7U4G

[a] Numbers in parentheses refer to the highest resolution shell.

[b] $R_{merge} = \Sigma |I_i - <I_i>| / \Sigma I_i$ where I_i = the intensity of the *i*th reflection and $<I_i>$ = mean intensity.

[c] $R_{work} = \Sigma |F_o - F_c| / \Sigma |F_o|$, where F_o and F_c are the observed and calculated structure factors, respectively.

[d] R_{free} was calculated as for R_{work}, but on a test set comprising 5% of the data excluded from refinement.

Supplementary Table 2. Primers for SD93 combinatorial mutagenesis experiment.

Primer name	Sequence
SD93lib-N387K-VF	5'-CGT ACG TCT CAC TGG TCC AAA CCT AAA TCC AAA TTG CAG-3'
SD93lib-VR	5'-CGT ACG TCT CAA AGC ACT TCC ATC AGT CAT TAC TAC TGT-3'
SD93lib-E248G-R249K-F	5'-CGT ACG TCT CAG CTT CAG RAA RAG CTG ATA CTA AAA TAC TAT TCA TTG AGG AGG GGA AA-3'
SD93lib-I265T-F	5'-TAA AAT ACT ATT CAT TGA GGA GGG GAA AAT CGT TCA TAY TAG CCC ATT GTC AGG AAG TGC-3'
SD93lib-336-346-5mut-F	5'-YAT TGC CKG RAT CCT AAC AAT GAG RAA GGG RGT CAT GGA GTG AAA GGC TGG GCC-3'
SD93lib-336-346-5mut-R	5'-ACY CCC TTY CTC ATT GTT AGG ATY CMG GCA ATR GCT ACT GCT GGA GCT GTC GTT-3'
SD93lib-E369K-R	5'-ACT TTG AAG GTT TCA TAA CCT GAG CGT AAC TYC TCG CTG ATC GTT CTT CCC ATC CA-3'
SD93lib-G381E-R	5'-CGT ACG TCT CAC CAG CCT YCA ATG ACT TTG AAG GTT TCA TAA CCT GAG CGT-3'
SD93lib-recover-F	5’-CAC TCT TTC CCT ACA CGA CGC TCT TCC GAT CTA GTA ATG ACT GAT GGA AGT GCT-3’
SD93lib-recover-R	5’-GAC TGG AGT TCA GAC GTG TGC TCT TCC GAT CTA CTT GCC TAT TTA TCT GCA ATT-3’

Supplementary Table 3. Primers for Bil69 combinatorial mutagenesis experiment.

Primer name	Sequence
Bil69lib-VR	5'-CGT ACG TCT CAA CTC CCA TCA GTC ATT ACT ACT GT-3'
Bil69lib-VF	5'-CGT ACG TCT CAG CTC AGG TTA TGA AAC TTT CAA AG-3'
Bil69lib-R249K-F	5'-CGT ACG TCT CAG AGT GCT TCA GGG ARA GCC GAT ACT AGA ATA CTA TTC ATT-3'
Bil69lib-D286G-R	5'-CCT ATT AGA GCC TTT CCA GTT GTC TCT GCA GAT ACA TCT GAC GYC AGG ATA TCG AGG ATA-3'
Bil69lib-I302V-M307V-R	5'-CTA TAA TCT TTC AYA TTT ATG TCT ACG AYG GGC CTA TTA GAG CCT TTC CAG TTG TCT CTG-3'
Bil69lib-I302V-M307V-F	5'-CCC RTC GTA GAC ATA AAT RTG AAA GAT TAT AGC ATT GAT-3'
Bil69lib-D329N-K334S-1-R	5'-CTG CAA TGG CTC TTG CTA GAT CTG TCG TYG TTT CTA GGT GTG TCG CCA AC-3'
Bil69lib-D329N-K334S-2-R	5'-CTG CAA TGG CTA CTG CTA GAT CTG TCG TYG TTT CTA GGT GTG TCG CCA AC-3'
Bil69lib-D329N-K334S-1-F	5'-RAC GAC AGA TCT AGC AAG AGC CAT TGC AGG AAT CCT AAC AAT GAG AGA GG-3'
Bil69lib-D329N-K334S-2-F	5'-RAC GAC AGA TCT AGC AGT AGC CAT TGC AGG AAT CCT AAC AAT GAG AGA GG-3'
Bil69lib-N356D-R	5'-TTG CTG ATC GTT CTT CCC ATC CAC ACG TCA TTT CCA TYG TCA AAG GCC CAG CCT-3'
Bil69lib-L370S-R	5'-CGT ACG TCT CAG AGC GTR AAT CCT TGC TGA TCG TTC TTC CCA TCC ACA CGT-3'
Bil69lib-recover-F	5'-CAC TCT TTC CCT ACA CGA CGC TCT TCC GAT CTA CAG TAG TAA TGA CTG ATG GGA GT-3'
Bil69lib-recover-R	5'-GAC TGG AGT TCA GAC GTG TGC TCT TCC GAT CTC TTT GAA AGT TTC ATA ACC TGA GC-3'

a

```
HK68  MNPNQKIITIGSVSLTIATVCFLMQIAILVTTVTLHFKQYECDSPASNQVMPCEPIIIERNITEIVYLNNTTIEKEICPK 80
Vic11 MNPNQKIITIGSVSLTISTICFFMQIAILITTVTLHFKQYEFNSPPNNQVMLCEPTIIERNITEIVYLTNTTIEKEICPK 80

HK68  VVEYRNWSKPQCQITGFAPFSKDNSIRLSAGGDIWVTREPYVSCDHGKCYQFALGQGTTLDNKHSNDTIHDRIPHRTLLM 160
Vic11 PAEYRNWSKPQCGITGFAPFSKDNSIRLSAGGDIWVTREPYVSCDPDKCYQFALGQGTTLNNVHSNNTVRDRTPYRTLLM 160

HK68  NELGVPFHLGTRQVCIAWSSSSCHDGKAWLHVCITGDDKNATASFIYDGRLVDSIGSWSQNILRTQESECVCINGTCTVV 240
Vic11 NELGVPFHLGTKQVCIAWSSSSCHDGKAWLHVCITGDDKNATASFIYNGRLVDSVVSWSKEILRTQESECVCINGTCTVV 240

HK68  MTDGSASGRADTRILFIEEGKIVHISPLSGSAQHVEECSCYPRYPGVRCICRDNWKGSNRPVVDINMEDYSIDSSYVCSG 320
Vic11 MTDGSASGKADTKILFIKEGKIVHTSTLSGSAQHVEECSCYPRYPGVRCVCRDNWKGSNRPIVDINIKDHSIVSSYVCSG 320

                       336                                                387
HK68  LVGDTPRNDDRSSNSNCRNPNNERGNQGVKGWAFDNGDDVWMGRTISKDLRSGYETFKVIGGWSTPNSKSQINRQVIVDS 400
Vic11 LVGDTPRKTDSSSSSHCLDPNNEEGGHGVKGWAFDDGNDVWMGRTINETSRLGYETFKVIEGWSNPKSKLQINRQVIVDR 400

HK68  DNRSGYSGIFSVEGKSCINRCFYVELIRGRKQETRVWWTSNSIVVFCGTSGTYGTGSWPDGANINFMPI 469
Vic11 GDRSGYSGIFSVEGKSCINRCFYVELIRGRKEETEVLWTSNSIVVFCGTSGTYGTGSWPDGADLNLMPI 469
```

b

```
Mos99 MNPNQKIITIGSVSLTIATICFLMQIAILVTTVTLHFKQYECNSPPNNQVMLCEPTIIERNITEIVYLTNTTIEKEICPK 80
Wy03  MNPNQKIITIGSVSLTISTICFFMQIAILITTVTLHFKQYEFNSPPNNQVMLCEPTIIERNITEIVYLTNTTIEKEICPK 80

Mos99 LAEYRNWSKPQCNITGFAPFSKDNSIRLSAGGDIWVTREPYVSCDPDKCYQFALGQGTTLNNGHSNDTVHDRTPYRTLLM 160
Wy03  LAEYRNWSKPQCNITGFAPFSKDNSIRLSAGGDIWVTREPYVSCDPDKCYQFALGQGTTLNNVHSNDTVHDRTPYRTLLM 160

Mos99 NELGVPFHLGTKQVCIAWSSSSCHDGKAWLHVCVTGDDENATASFIYNGRLVDSIGSWSKKILRTQESECVCINGTCTVV 240
Wy03  NELGVPFHLGTKQVCIAWSSSSCHDGKAWLHVCVTGDDENATASFIYNGRLVDSIVSWSKKILRTQESECVCINGTCTVV 240

Mos99 MTDGSASGKADTKILFIEEGKIVHTSPLSGSAQHVEECSCYPRYPGVRCVCRDNWKGSNRPIVDINVKDYSIVSSYVCSG 320
Wy03  MTDGSASGKADTKILFIEEGKIVHTSTLSGSAQHVEECSCYPRYPGVRCVCRDNWKGSNRPIVDINIKDYSIVSSYVCSG 320

                                                                         385
Mos99 LVGDTPRKNDSSSSSHCLDPNNEEGGHGVKGWAFDDGNDVWMGRTISEKLRSGYETFKVIEGWSKPNSKLQINRQVIVDR 400
Wy03  LVGDTPRKNDSSSSSHCLDPNNEEGGHGVKGWAFDDGNDVWMGRTISEKLRSGYETFKVIEGWSNPNSKLQINRQVIVDR 400

Mos99 GNRSGYSGIFSVEGKSCINRCFYVELIRGRKQETEVLWTSNSIVVFCGTSGTYGTGSWPDGADINLMPI 469
Wy03  GNRSGYSGIFSVEGKSCINRCFYVELIRGRKQETEVLWTSNSIVVFCGTSGTYGTGSWPDGADINLMPI 469
```

c

```
SD93  MNPNQKIITIGSVTLTIATICFLMQIAILVTTVTLHFKQYECNSPPNNQVMLCEPTIIERNITEIVYLTNTTIEKEICPK 80
Mos99 MNPNQKIITIGSVSLTIATICFLMQIAILVTTVTLHFKQYECNSPPNNQVMLCEPTIIERNITEIVYLTNTTIEKEICPK 80

SD93  LAEYRNWSKPQCKITGFAPFSKDNSIRLSAGGDIWVTREPYVSCDPGKCYQFALGQGTTLNNRHSNDTVHDRTPYRTLLM 160
Mos99 LAEYRNWSKPQCNITGFAPFSKDNSIRLSAGGDIWVTREPYVSCDPDKCYQFALGQGTTLNNGHSNDTVHDRTPYRTLLM 160

SD93  NELGVPFHLGTKQVCIAWSSSSCHDGKAWLHVCVTGHDENATASFIYDGRLVDSIGSWSKNILRTQESECVCINGTCTVV 240
Mos99 NELGVPFHLGTKQVCIAWSSSSCHDGKAWLHVCVTGDDENATASFIYNGRLVDSIGSWSKKILRTQESECVCINGTCTVV 240

           248&249         265
SD93  MTDGSASERADTKILFIEEGKIVHISPLSGSAQHVEECSCYPRYPGVRCVCRDNWKGSNRPIVDINVKDYSIVSSYVCSG 320
Mos99 MTDGSASGKADTKILFIEEGKIVHTSPLSGSAQHVEECSCYPRYPGVRCVCRDNWKGSNRPIVDINVKDYSIVSSYVCSG 320

                      336 338&339 344 346                    369          381
SD93  LVGDTPRKNDSSSSSYCRNPNNEKGSHGVKGWAFDDGNDVWMGRTISEELRSGYETFKVIGGWSKPNSKLQINRQVIVDR 400
Mos99 LVGDTPRKNDSSSSSHCLDPNNEEGGHGVKGWAFDDGNDVWMGRTISEKLRSGYETFKVIEGWSKPNSKLQINRQVIVDR 400

SD93  GNRSGYSGIFSVEGKSCINRCFYVELIRGRKQETEVWWTSNSIVVFCGTSGTYGTGSWPDGGDINLMPI 469
Mos99 GNRSGYSGIFSVEGKSCINRCFYVELIRGRKQETEVLWTSNSIVVFCGTSGTYGTGSWPDGADINLMPI 469
```

d

```
Bil69 MNPNQKIITIGSVSLIIATVCFLMQIAILVTTVTLHFKQHECDSPSSNQVMLCEPIIIERNITEIVYLNNTTIEKETCPK 80
Bil71 MNPNQKIITIGSISLTIATICFLMQIAILVTTVTLHFKQYECDSPANNQVMPCEPIIIERNITEIVYLTNTTIEKEICPK 80

Bil69 LVEYRNWSKPQCKITGFAPFSKDNSIRLSAGGDIWVTREPYVSCDPGKCYQFALGQGTTLDNKHSNDTIHDRIPHRTLLM 160
Bil71 LVEYRNWSKPQCKITGFAPFSKDNSIRLSAGGDIWVTREPYVSCDPGKCYQFALGQGTTLDNKHSNDTIHDRIPHRTLLM 160

Bil69 NELGVPFHLGTRQVCIAWSSSSCHDGKAWLHVCVTGDDKNATASFIYDGRLVDSIGSWSQNILRTQESECVCINGTCTVV 240
Bil71 NELGVPFHLGTRQVCIAWSSSSCHDGKAWLHVCVTGDDKNATASFIYDGRLVDSIGSWSQNILRTQESECVCINGTCTVV 240

           249                                  286            302  307
Bil69 MTDGSASGRADTRILFIEEGKIVHISPLSGSAQHVEECSCYPRYPDVRCICRDNWKGSNRPIVDINMKDYSIDSSYVCSG 320
Bil71 MTDGSASGKADTRILFIEEGKIVHISPLSGSAQHVEECSCYPRYPGVRCICRDNWKGSNRPVVDINVKDYSIDSSYVCSG 320

            329   334 336                 356            370
Bil69 LVGDTPRNDDRSSSKSNCRNPNNERGNHGVKGWAFDNGNDVWMGRTISKDLRSGYETFKVIGGWSTPNSKSQINRQVIVDS 400
Bil71 LVGDTPRNNDRSSSSSYCRNPNNERGNHGVKGWAFDDGNDVWMGRTISKDSRSGYETFKVIGGWSTPNSKSQINRQVIVDS 400

Bil69 DNRSGYSGIFSVEGKSCINRCFYVELIRGREQETRVWWTSNSIVVFCGTSGTYGTGSWPDGANINFMPI 469
Bil71 DNRSGYSGIFSVEGKSCINRCFYVELIRGREQETRVWWTSNSIVVFCGTSGTYGTGSWPDGADINLMPI 469
```

Supplementary Figure 1. Sequence alignment of influenza virus H3N2 neuraminidase (NA). Alignments of NA amino acid sequences between **(a)** HK68 and Vic11, **(b)** Mos99 and Wy03 **(c)** SD93 and Mos99, and **(d)** Bil69 and Bil71. Residues that are not conserved between sequences are highlighted in pink. Mutations of interest are labeled in red. NA head domain is from residues 82 to 469.

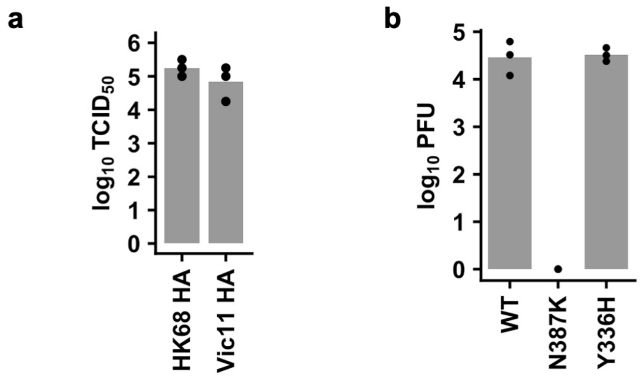

Supplementary Figure 2. Fitness effects of NA mutations are largely independent of other segments. (a) The replication fitness of viruses that carry HK68 NA but different HAs (HK68 HA and Vic11 HA) was examined by a virus rescue experiment. Virus titer was measured by $TCID_{50}$. **(b)** The replication fitness of NA N387K and NA Y336H in authentic H3N2 A/Udorn/1972 (all eight segments were from A/Udorn/72) was examined by a virus rescue experiment. Virus titer was measured by plaque forming units (PFU). Each bar represents the mean of three independent biological replicates. Each dot represents one biological replicate. Source data are provided as a Source Data file.

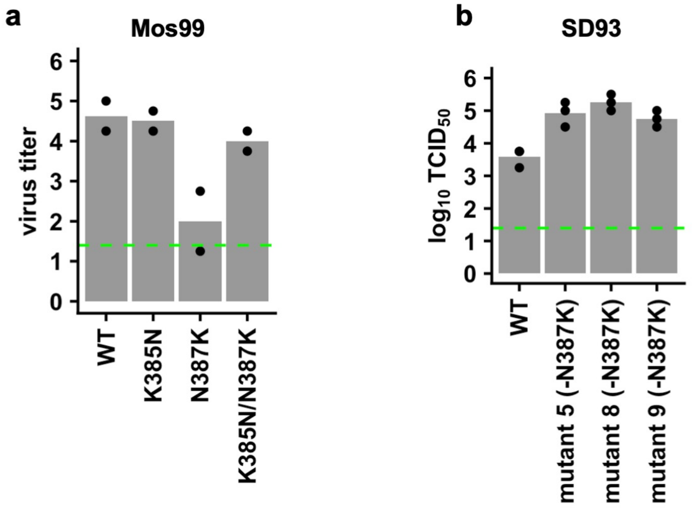

Supplementary Figure 3. Virus replication fitness of permissive mutations for N387K. The replication fitness of different **(a)** Mos99 NA mutants, and **(b)** SD93 NA mutants was examined by a virus rescue experiment. Mutant 5 (-N387K): E248G/R249K/Y336N/K344E/E369K, Mutant 8 (-N387K): E248G/R249K/Y336N/K344E/E369K/K385N, Mutant 9 (-N387K): E248G/R249K/Y336H/K344E/E369K/K385N. Virus titer was measured by $TCID_{50}$. The green dashed line represents the lower detection limit. Each bar represents the mean of **(a)** two or **(b)** three independent biological replicates. Each dot represents one biological replicate. Source data are provided as a Source Data file.

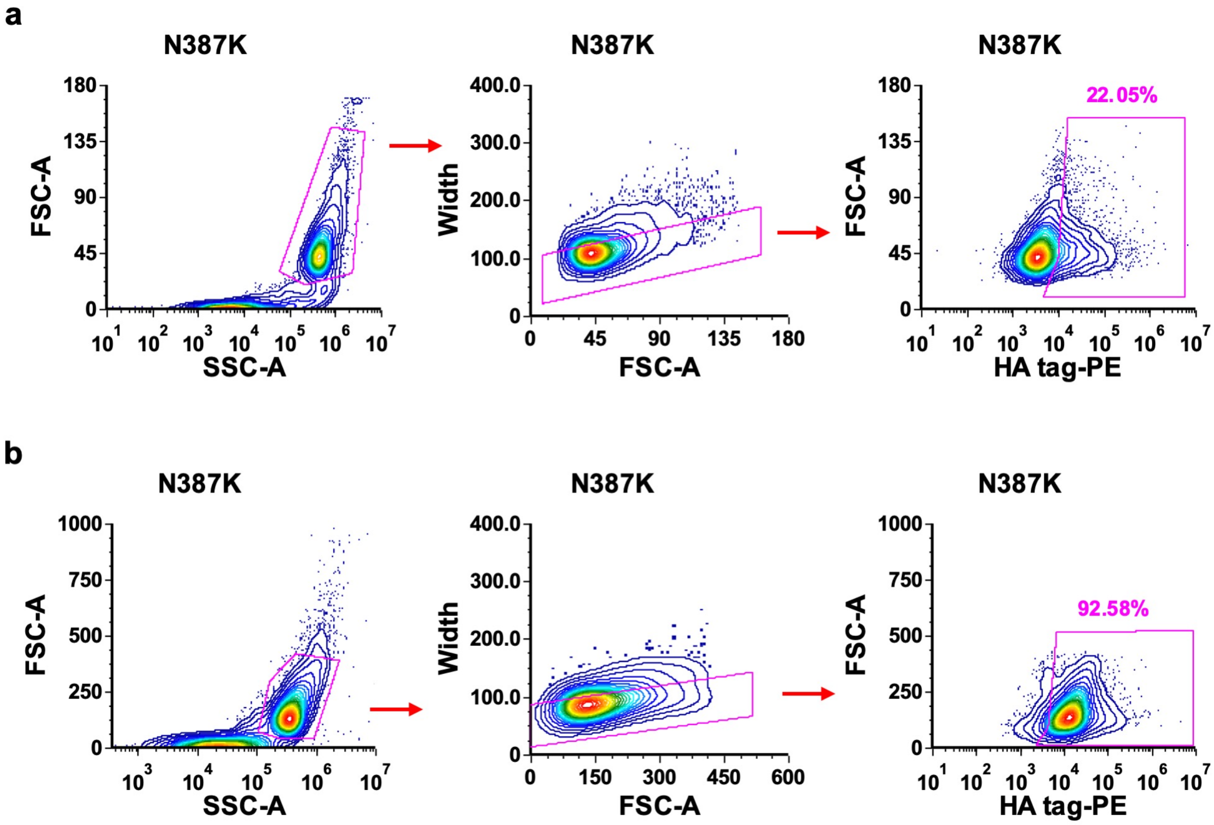

Supplementary Figure 4. Gating strategies for flow cytometry in this study. Gating strategy for **(a)** surface expression, and **(b)** intracellular expression of NA. Mos99 N387K is shown as an example.

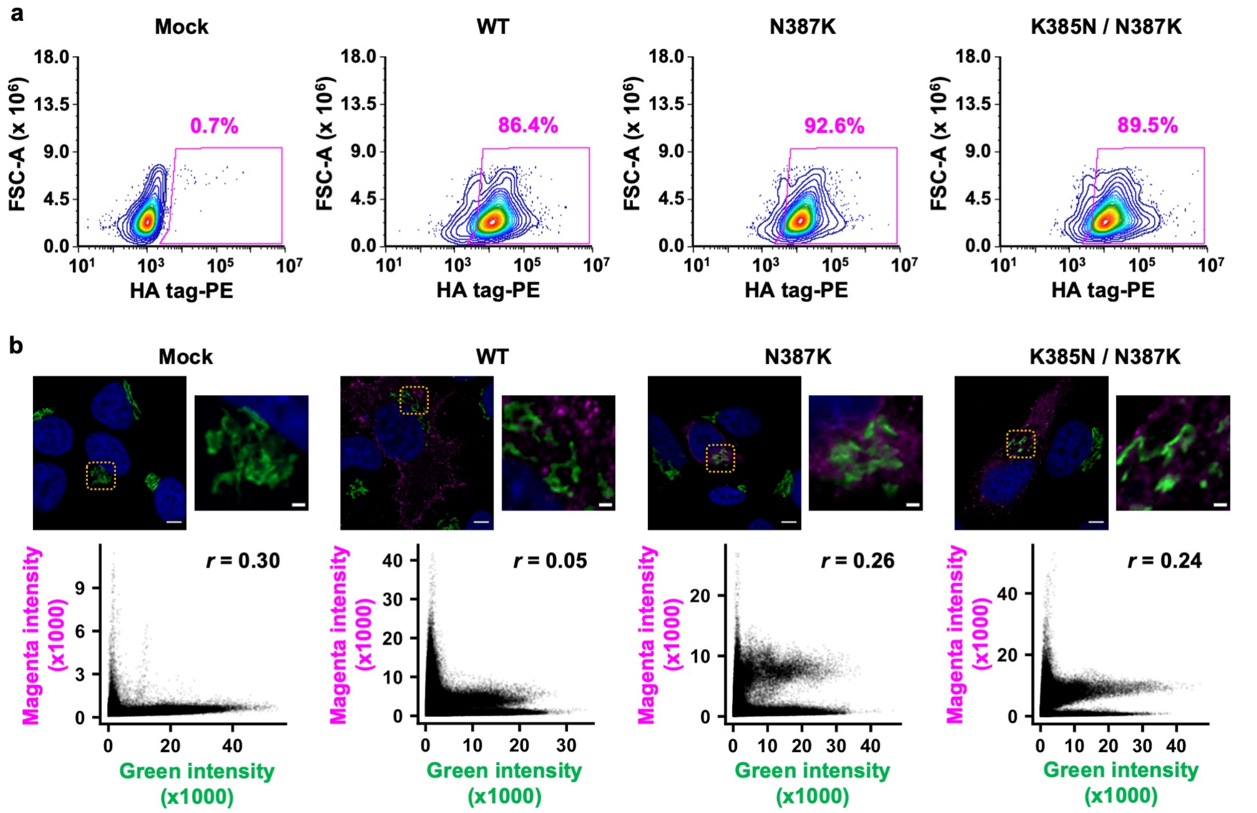

Supplementary Figure 5. Cellular expression and localization of Mos99 NA mutants. (a) Flow cytometry analysis of 293T cells that transiently expressed HA-tagged Mos99 NA mutants. **(b)** Confocal microscopy analysis of HA-tagged Mos99 NA mutants. Blue (DAPI), Green (GM130, Golgi), Magenta (NA). The orange box highlights the zoomed-in region, which is shown on the left. Scale bar for large image is 5 μm and scale bar for zoomed-in image is 2 μm. Below each micrograph is a cytofluorogram, in which each data point represents a pixel. The Pearson correlation coefficient between the green intensity (GM130, Golgi) and magenta intensity (NA) across all pixels in each image is indicated. The result of a representative experiment out of two independent biological replicates is shown.

a

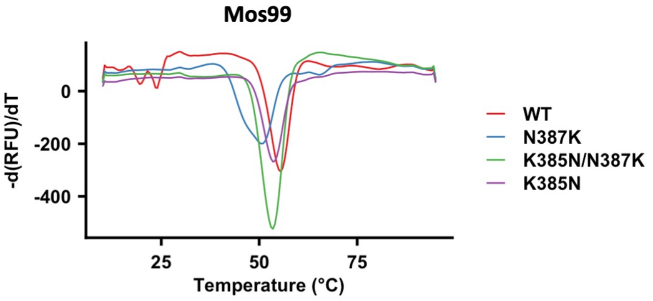

Variant	T_m (°C)
WT	55.5
N387K	50.7
K385N/N387K	53.5
K385N	53.0

b

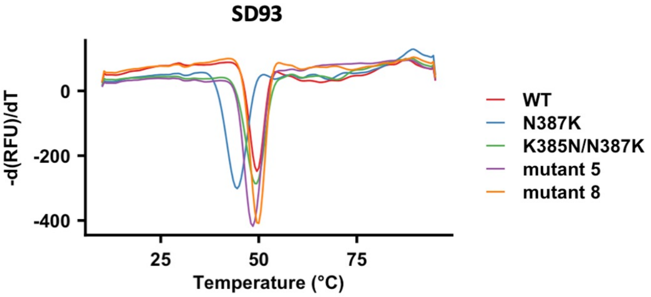

Variant	T_m (°C)
WT	49.5
N387K	44.5
K385N/N387K	49.3
mutant 5	48.5
mutant 8	49.8

c

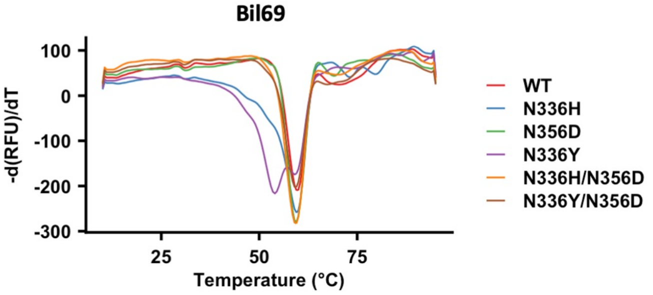

Variant	T_m (°C)
WT	59.6
N336H	59.5
N356D	59.7
N336Y	53.9
N336H/N356D	59.3
N336Y/N356D	59.3

Supplementary Figure 6. Measuring the thermal stability of different NA mutants using SYPRO orange dye-based thermal shift assay. The first differential curves for the relative fluorescence unit (RFU) with respect to temperature are shown for **(a)** Mos99 NA WT and mutants, **(b)** SD93 NA WT and mutants, and **(c)** Bil69 NA WT and mutants. For SD93, mutant 5 represents E248G/R249K/Y336N/K344E/E369K/N387K, whereas mutant 8 represents E248G/R249K/Y336N/K344E/E369K/K385N/N387K. The melting temperature (T_m), which corresponds to the lowest point of the first derivative −d(RFU)/dT, is listed for each variant. The reported T_m is an average of six independent biological replicates. **(a-c)** The result of a representative experiment out of six independent biological replicates is shown.

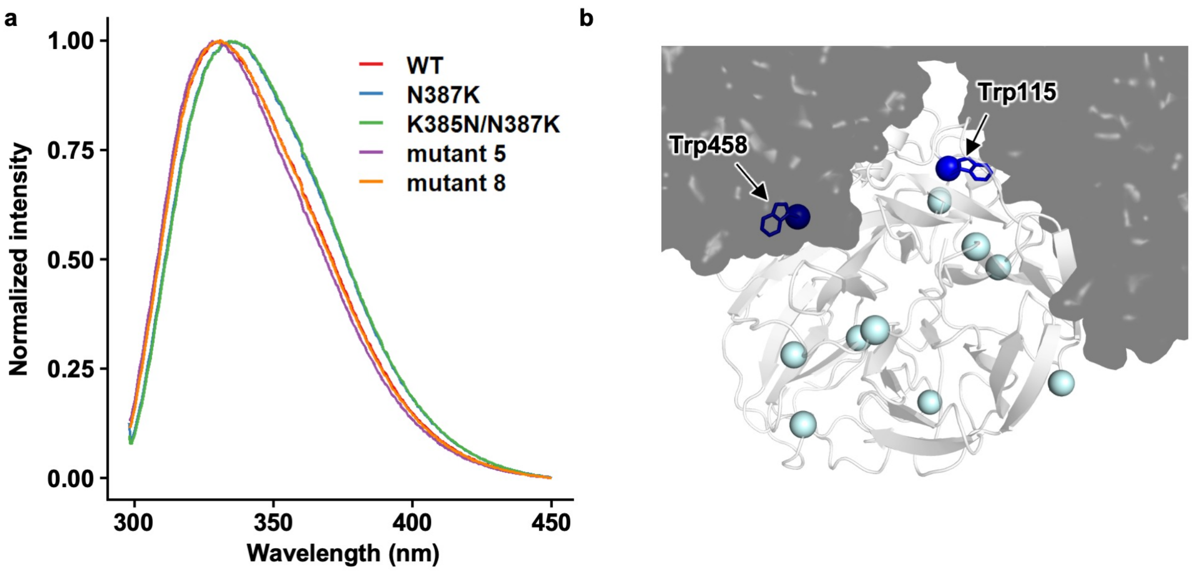

Supplementary Figure 7. Tryptophan emission spectrum of SD93 NA WT and mutants. (a) Normalized steady-state emission spectrum of SD93 NA WT and mutants, using λ_{exc} = 295 nm. Of note, the blue (N387K) and green (K385N/N387K) curves almost completely overlap. Mutant 5 represents E248G/R249K/Y336N/K344E/E369K/N387K, whereas mutant 8 represents E248G/R249K/Y336N/K344E/E369K/K385N/N387K. The result of a representative experiment out of two is shown. **(b)** Two out of 12 tryptophans in the head domain of SD93 NA are located at the protomer-protomer interface. Tryptophans are shown as spheres on one protomer that is in white cartoon representation, while the other three protomers are show as semitransparent black surface. The two tryptophans at the protomer-protomer interface, namely Trp115 and Trp458, are shown in blue. Other tryptophans are in cyan.
