# Supplementary material for: Prevalence and mechanisms of evolutionary contingency in human influenza H3N2 neuraminidase
Source: Nat Commun. 2022 Oct 28;13:6443. doi: 10.1038/s41467-022-34060-8 (PMC9616408; doi:10.1038/s41467-022-34060-8)
Supplement: Supplementary file 3 — Reporting Summary [file 41467_2022_34060_MOESM3_ESM.pdf]

nature portfolio

Corresponding author(s): Nicholas C. Wu

Last updated by author(s): Oct 10, 2022

Reporting Summary

Nature Portfolio wishes to improve the reproducibility of the work that we publish. This form provides structure for consistency and transparency in reporting. For further information on Nature Portfolio policies, see our Editorial Policies and the Editorial Policy Checklist.

Statistics

For all statistical analyses, confirm that the following items are present in the figure legend, table legend, main text, or Methods section.

n/a	Confirmed	
☐	☒	The exact sample size (n) for each experimental group/condition, given as a discrete number and unit of measurement
☐	☒	A statement on whether measurements were taken from distinct samples or whether the same sample was measured repeatedly
☒	☐	The statistical test(s) used AND whether they are one- or two-sided<br>*Only common tests should be described solely by name; describe more complex techniques in the Methods section.*
☒	☐	A description of all covariates tested
☒	☐	A description of any assumptions or corrections, such as tests of normality and adjustment for multiple comparisons
☒	☐	A full description of the statistical parameters including central tendency (e.g. means) or other basic estimates (e.g. regression coefficient) AND variation (e.g. standard deviation) or associated estimates of uncertainty (e.g. confidence intervals)
☒	☐	For null hypothesis testing, the test statistic (e.g. F, t, r) with confidence intervals, effect sizes, degrees of freedom and P value noted<br>*Give P values as exact values whenever suitable.*
☒	☐	For Bayesian analysis, information on the choice of priors and Markov chain Monte Carlo settings
☒	☐	For hierarchical and complex designs, identification of the appropriate level for tests and full reporting of outcomes
☐	☒	Estimates of effect sizes (e.g. Cohen's d, Pearson's r), indicating how they were calculated

Our web collection on statistics for biologists contains articles on many of the points above.

Software and code

Policy information about availability of computer code

Data collection	Data were analyzed using FCS Express 6 software (De Novo Software). Cytofluorogram data and Pearson correlation coefficients of transfected cells were calculated with fluorescence intensity thresholds automatically adjusted using the JACoP plug-in. The structure was solved by molecular replacement using Phaser-MR included in the Phenix suite, using as PDB 2AEP as the replacement model. The structure was further refined using REFMAC5 and was manually built in COOT. Ramachandran statistics were calculated using MolProbity.
Data analysis	Custom python scripts for analyzing the deep mutational scanning data have been deposited to https://github.com/nicwulab/N2_evol_contingency.

For manuscripts utilizing custom algorithms or software that are central to the research but not yet described in published literature, software must be made available to editors and reviewers. We strongly encourage code deposition in a community repository (e.g. GitHub). See the Nature Portfolio guidelines for submitting code & software for further information.

Data

Policy information about availability of data

All manuscripts must include a data availability statement. This statement should provide the following information, where applicable:

- Accession codes, unique identifiers, or web links for publicly available datasets
- A description of any restrictions on data availability
- For clinical datasets or third party data, please ensure that the statement adheres to our policy

Raw sequencing data generated in this study have been deposited in the NIH Short Read Archive under accession number: BioProject PRJNA790468. The X-ray coordinates and structure factors have been deposited in the RCSB Protein Data Bank under accession codes 7U4E, 7U4F, and 7U4G. Source data are provided with this paper.

Field-specific reporting

Please select the one below that is the best fit for your research. If you are not sure, read the appropriate sections before making your selection.

- [x] Life sciences
- [ ] Behavioural & social sciences
- [ ] Ecological, evolutionary & environmental sciences

For a reference copy of the document with all sections, see nature.com/documents/nr-reporting-summary-flat.pdf

Life sciences study design

All studies must disclose on these points even when the disclosure is negative.

Sample size	Not applicable
Data exclusions	No data were excluded
Replication	Biological replicates were included
Randomization	Not applicable
Blinding	Not applicable

Reporting for specific materials, systems and methods

We require information from authors about some types of materials, experimental systems and methods used in many studies. Here, indicate whether each material, system or method listed is relevant to your study. If you are not sure if a list item applies to your research, read the appropriate section before selecting a response.

Materials & experimental systems

n/a	Involved in the study	
☐	☒	Antibodies
☐	☒	Eukaryotic cell lines
☒	☐	Palaeontology and archaeology
☒	☐	Animals and other organisms
☒	☐	Human research participants
☒	☐	Clinical data
☒	☐	Dual use research of concern

Methods

n/a	Involved in the study	
☒	☐	ChIP-seq
☐	☒	Flow cytometry
☒	☐	MRI-based neuroimaging

Antibodies

Antibodies used	PE anti-HA.11 clone 16B12 (BioLegend, Cat # 901518)<br>Mouse anti-HA tag (Invitrogen, Cat # 26183)<br>Rabbit anti-calnexin (Invitrogen, Cat # PA5-34754)<br>Rabbit anti-GM130 (ProteinTech, Cat # 11308-1-AP)<br>Alexa Fluor 488 donkey anti-rabbit IgG (Abcam, Cat # ab150073)<br>Alexa Fluor 555 donkey anti-mouse IgG (Invitrogen, Cat # A-31570)
Validation	PE anti-HA.11 clone 16B12: FC, ICFC<br>Mouse anti-HA tag: WB, ICC/IF, IP<br>Rabbit anti-calnexin: WB, IHC (P), ICC/IF, IP<br>Rabbit anti-GM130: WB, IHC, IF<br>Alexa Fluor 488 donkey anti-rabbit IgG: ICC/IF, Flow Cyt, IHC-P, ELISA, IHC-Fr<br>Alexa Fluor 555 donkey anti-mouse IgG: IHC, ICC/IF

Eukaryotic cell lines

Policy information about cell lines

Cell line source(s)	MDCK-SIAT1 cells (Sigma-Aldrich, Cat#: 05071502-1VL)<br>Sf9 cells (ATCC, Cat#: CRL-1711)
Authentication	None of the cell lines used were authenticated

Mycoplasma contamination	All cell lines tested negative for mycoplasma contamination
Commonly misidentified lines (See ICLAC register)	*Name any commonly misidentified cell lines used in the study and provide a rationale for their use.*

Flow Cytometry

Plots

Confirm that:

- [x] The axis labels state the marker and fluorochrome used (e.g. CD4-FITC).
- [x] The axis scales are clearly visible. Include numbers along axes only for bottom left plot of group (a 'group' is an analysis of identical markers).
- [x] All plots are contour plots with outliers or pseudocolor plots.
- [x] A numerical value for number of cells or percentage (with statistics) is provided.

Methodology

Sample preparation

4x10^5 HEK 293T cells were seeded on each well of 6-well plates and grown overnight at 37 °C and 5% CO_2 in a humidified incubator. Cells were transfected with 2 µg of phCMV3 plasmids encoding the C-terminal HA-tagged NA from the strain of interest using Lipofectamine 2000 (Thermo Fisher Scientific) according to the manufacturer's instructions. At 24 h post-transfection, medium was discarded and cells were washed once with PBS. Cells were subsequently detached with versene (Thermo Fisher Scientific) and resuspended in fluorescence-activated single cell sorting (FACS) buffer (50 mM EDTA, 2% v/v FBS in DMEM with high glucose and HEPES, without phenol red). Cells were then pelleted via centrifugation at 300 xg for 5 min at 4 °C and the supernatant was aspirated. In the subsequent steps, cells and all reagents were kept on ice.

For surface staining, cell pellets were resuspended in 1 mL FACS buffer. Cells were incubated with 1 µg/mL PE-anti HA.11 (BioLegend) at 4 °C with rocking for 1 h. Subsequently, cells were pelleted via centrifugation at 300 xg for 5 min at 4 °C and the supernatant was aspirated. Cells were washed with 1 mL FACS buffer, pelleted, and resuspended in 1 mL FACS buffer.

For intracellular staining, cell pellets were fixed in 300 µL of 1% v/v paraformaldehyde (in PBS) and incubated at 4 °C with rocking for 30 min. Cells were pelleted, washed with 1 mL FACS buffer, permeabilized in 300 µL of 0.1% v/v Triton X-100 (in PBS) and incubated at 4 °C with rocking for 30 min. Cells were pelleted, washed with 1 mL FACS buffer and incubated with 1 µg/mL PE-anti HA.11 (BioLegend) at 4 °C with rocking for 1 h. Then, cells were pelleted, washed with 1 mL FACS buffer, and resuspended in 1 mL FACS buffer.

Instrument	Cells were analyzed using an Accuri C6 flow cytometer (BD Biosciences) with a 488 nm laser and a 585/40 bandpass filter.
Software	Data were collected using Accuri C6 software. Data were analyzed using FCS Express 6 software (De Novo Software).
Cell population abundance	10^4 singlets were collected for each sample.
Gating strategy	*Describe the gating strategy used for all relevant experiments, specifying the preliminary FSC/SSC gates of the starting cell population, indicating where boundaries between "positive" and "negative" staining cell populations are defined.*

- [x] Tick this box to confirm that a figure exemplifying the gating strategy is provided in the Supplementary Information.
